# Colossal flexoresistance in dielectrics

**DOI:** 10.1038/s41467-020-16207-7

**Published:** 2020-05-22

**Authors:** Sung Min Park, Bo Wang, Tula Paudel, Se Young Park, Saikat Das, Jeong Rae Kim, Eun Kyo Ko, Han Gyeol Lee, Nahee Park, Lingling Tao, Dongseok Suh, Evgeny Y. Tsymbal, Long-Qing Chen, Tae Won Noh, Daesu Lee

**Affiliations:** 1 Center for Correlated Electron Systems, Institute for Basic Science (IBS), Seoul, 08826 Korea; 2 Department of Physics and Astronomy, Seoul National University, Seoul, 08826 Korea; 3 Department of Materials Science and Engineering, The Pennsylvania State University, University Park, PA 16802 USA; 4 Department of Physics and Astronomy & Nebraska Center for Materials and Nanoscience, University of Nebraska, Lincoln, NE 68588 USA; 5 Department of Physics, Soongsil University, Seoul, 07027 Korea; 6 Department of Energy Science, Sungkyunkwan University, Suwon, 16419 Korea; 7 Department of Physics, Pohang University of Science and Technology (POSTECH), Pohang, 37673 Korea; 8 Asia Pacific Center for Theoretical Physics, Pohang, 37673 Korea

**Keywords:** Electronic properties and materials, Ferroelectrics and multiferroics

## Abstract

Dielectrics have long been considered as unsuitable for pure electrical switches; under weak electric fields, they show extremely low conductivity, whereas under strong fields, they suffer from irreversible damage. Here, we show that flexoelectricity enables damage-free exposure of dielectrics to strong electric fields, leading to reversible switching between electrical states—insulating and conducting. Applying strain gradients with an atomic force microscope tip polarizes an ultrathin film of an archetypal dielectric SrTiO_3_ via flexoelectricity, which in turn generates non-destructive, strong electrostatic fields. When the applied strain gradient exceeds a certain value, SrTiO_3_ suddenly becomes highly conductive, yielding at least around a 10^8^-fold decrease in room-temperature resistivity. We explain this phenomenon, which we call the colossal flexoresistance, based on the abrupt increase in the tunneling conductance of ultrathin SrTiO_3_ under strain gradients. Our work extends the scope of electrical control in solids, and inspires further exploration of dielectric responses to strong electromechanical fields.

## Abstract (web summary)

Manipulating the electric state of large band gap dielectrics without any damage is quite challenging. Here, the authors demonstrate by mechanically introducing strain gradients that large electric fields are generated via flexoelectric interactions, resulting in a reversible Zener breakdown in SrTiO_3_, changing the resistivity by 10^8^.

## Introduction

Controlling electron dynamics in solids has opened avenues for fascinating physical phenomena^1–3^ and has formed the basis of electronic applications. In semiconductors with a relatively small but nonzero bandgap, applying moderate electric fields could switch their electrical state, i.e., from insulator to conductor, which makes them a building block for contemporary digital electronics. In dielectrics with a large bandgap, controlling their electrical states is quite complicated, as it usually involves a combination of intrinsic and extrinsic processes. Zener^4^ predicted that strong electric fields (≥10^9^ V m^−1^) could intrinsically lead to electrical breakdown in dielectrics through tunneling processes across the valence and conduction bands. As this dielectric breakdown naturally guarantees the largest and fastest electrical response, recent works have aimed to realize it by applying strong femtosecond fields^1,2^. Under strong static fields, however, the dielectric breakdown has been unavoidably subject to extrinsic effects^5,6^, such as Joule heating and irreversible damage. This situation complicates our understanding of the intrinsic mechanism of dielectric breakdown and limits device application.

Here, we demonstrate that electrical states in dielectrics can be controlled by means of depolarization field induced by flexoelectric polarization. By applying the strain gradients from a conductive scanning probe tip, we simultaneously polarize and measure the current across the film. Above the certain critical strain gradients, the current–voltage (*I*–*V*) characteristic changes from tunneling-like to linear, which indicates the change of the electrical state from insulating to conducting. We explain this phenomena with a modulation of band structure due to the electrostatic field induced by flexoelectricity.

## Results

### Concept of flexoelectric control of electrical states in dielectrics

**Fig. 1:**
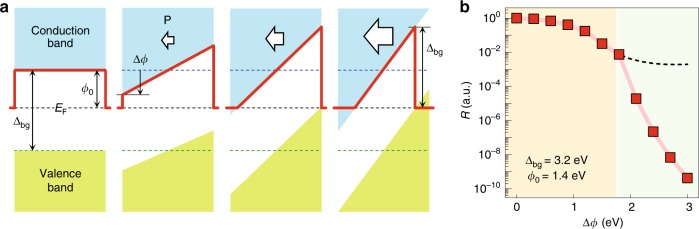
Colossal decrease of resistivity in highly polarized ultrathin dielectrics. **a** Schematic diagram of the potential energy profiles across SrTiO_3_ (STO) with increasing flexoelectric polarization (**P**; white arrow). Red solid lines and black dashed lines indicate the effective tunnel barrier and Fermi level, respectively. Blue and green dashed lines indicate the conduction band minimum and valence band maximum for **P** = 0, respectively. **b** Resistance as a function of ∆*φ*, obtained by calculating tunneling conductance through a Wentzel–Kramers–Brillouin (WKB) approximation. We normalize the resistance by the value at ∆*φ* = 0, and assume the bandgap ∆_bg_, original barrier height *φ*_0_, and original barrier width *d*_0_ to be 3.2 eV, 1.4 eV, and 3.9 nm, respectively. At ∆*φ* = 1.8 eV, the valence band maximum crosses the Fermi level, which causes an abrupt reduction in the resistance. The black dashed line indicates the result obtained by neglecting the valence band contribution. Source data are provided as a Source data file.

To achieve intrinsic, static control of electrical states in dielectrics, we could utilize a non-destructive electrostatic field developed in ultrathin polar materials (Fig. 1a). When a polar material is sufficiently thin but still maintains polarization *P*, a depolarization field *E*_dep_ arises from the unscreened bound charges on its surface^7,8^:

1 $$E_{{\mathrm{dep}}} = - \frac{{P - \sigma _{\mathrm{S}}}}{\varepsilon },$$

where *σ*_S_ is the screening charge (e.g., by adjacent metal electrodes) and *ε* is the dielectric permittivity of the polar material. In the ultrathin limit, *σ*_S_ tends to zero^8^ and *E*_dep_ becomes increasingly saturated at *E*_dep_ = −*P*/*ε*, largely modifying the band structure (Fig. 1a). In particular, when the polarization exceeds a certain threshold, both the conduction band minimum and valence band maximum could cross the Fermi level, as confirmed in our first-principles calculation (Supplementary Fig. 1). In such a case, the tunnel-barrier width of ultrathin dielectrics would abruptly decrease, whereas the tunnel-barrier height remains fixed to the bandgap ∆_bg_ (Fig. 1a and Supplementary Fig. 2). This would result in a significant enhancement of tunneling conductance across ultrathin dielectrics, leading to a colossal decrease in electrical resistance, as predicted in our Wentzel–Kramers–Brillouin (WKB) simulation (Fig. 1b). Therefore, it would be of great interest to explore tunnel transport across a highly polarized ultrathin dielectric.

**Fig. 2:**
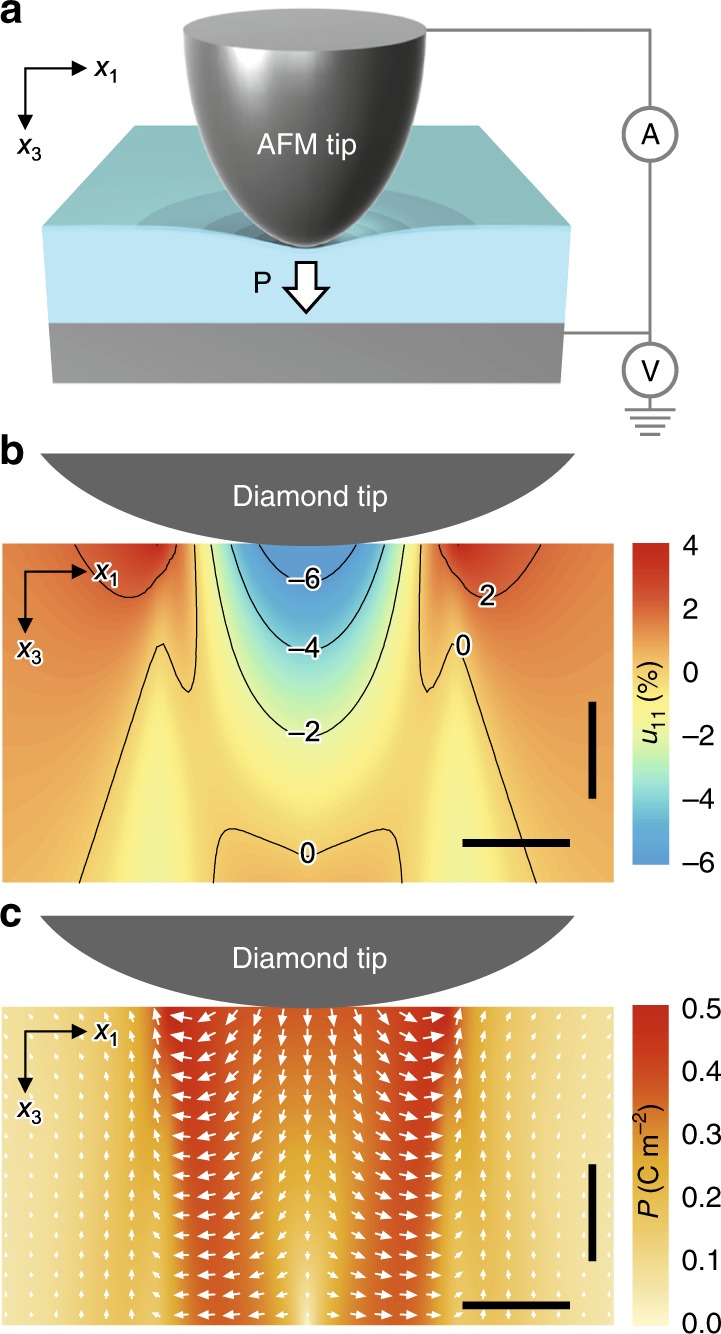
Mechanically induced large polarization in ultrathin dielectrics. **a** Schematic diagram of the experimental setup, illustrating the flexoelectric polarization (white arrow) generated by the atomic force microscope (AFM) tip pressing the surface of ultrathin dielectrics. While generating large strain gradients, we simultaneously measure the tunneling currents across the flexoelectrically polarized STO. **b**, **c** Phase-field simulations for the transverse strain *u*_11_ (**b**) and corresponding polarization distribution (**c**) in ultrathin STO under a representative tip loading force of 15 μN over a circular area ~13 nm in radius. Vertical and horizontal scale bars represent 1 nm and 10 nm, respectively. Source data are provided as a Source data file.

To this end, we can induce and stabilize large polarization in an ultrathin dielectric via flexoelectricity^9–20^. All dielectric materials polarize in response to strain gradients, as follows:

2 $$P = \varepsilon \cdot f_{{\mathrm{eff}}} \cdot \frac{{\partial u}}{{\partial x}},$$

where ∂*u*/∂*x* and *f*_eff_ are the strain gradient and effective flexocoupling coefficient, respectively. Applying loading forces through an atomic force microscope (AFM) tip (Fig. 2a) generates strain gradients as large as 10^7^ m^−1^ in ultrathin dielectrics^13,17–19^. Such giant strain gradient could then induce flexoelectric polarization, up to a few 0.1 Cm^−2^ (ref. ^19^), much larger than the polarization values typically attainable in ultrathin ferroelectrics^21,22^.

### Colossal flexoresistance in an archetypal dielectric SrTiO_3_

We choose SrTiO_3_ (STO) as a model dielectric system, as it shows enhanced flexocoupling strength at the nanoscale^19^, as well as a reasonably large bandgap of 3.2 eV. Importantly, furthermore, its conductivity responds negligibly to the applied strain itself (Supplementary Fig. 3)^23,24^, thereby maximizing the contribution from strain gradient-induced flexoelectricity. We prepare 10-unit-cell-thick (i.e., 3.9-nm thick) stoichiometric STO films on a (001)-oriented STO single crystal substrate, with a conductive SrRuO_3_ buffer layer (Supplementary Figs. 4 and 5). The stoichiometric homoepitaxial STO should remain paraelectric down to 0 K (ref. ^25^); however, under an AFM-tip loading force, it can become highly polarized via flexoelectricity^19^.

We then use contact mechanics analysis to simulate strain gradients and associated flexoelectric polarization in ultrathin STO under an AFM-tip loading force (see Methods). For the simulation, we adopt a diamond tip and assume a tip radius of curvature (*r*_tip_) of 100 nm. Note that the actual contact radius is estimated to be around 13 nm for the case of a 15 μN tip loading force, which is much smaller than the tip radius *r*_tip_. Figure 2b shows a simulated profile of transverse strain *u*_11_ under a representative tip loading force of 15 μN, revealing the large inhomogeneity of *u*_11_. The resulting transverse strain gradients ∂*u*_t_/∂*x*_3_ (i.e., =∂*u*_11_/∂*x*_3_ + ∂*u*_22_/∂*x*_3_) are as huge as a few 10^7^ m^−1^ (Supplementary Fig. 6); this giant strain gradients are attributable to AFM-tip-induced downward bending at the nanoscale. Our simulation also finds that those strain gradients induce large polarization in ultrathin STO via flexoelectricity, reaching up to 0.18 Cm^−2^ on average (Fig. 2c). When neglecting flexoelectricity, our simulation does not produce any polarization, confirming the flexoelectric nature of the induced polarization.

When such a large polarization remains preserved in an ultrathin dielectric, it could significantly modify the band structure of the dielectric, as predicted in Fig. 1. We estimate the threshold polarization *P*_th_ in ultrathin STO, above which both the conduction band minimum and valence band maximum cross the Fermi level (Fig. 1a):

3 $$\left| P_{\mathrm{th}} \right| \approx \varepsilon \cdot E_{{\mathrm{dep}},{\mathrm{th}}} = \varepsilon \cdot \frac{{\Delta}_{bg}}{{\it{e}} \cdot {\it{t}}},$$

where ∆_bg_ and *t* are the bandgap and thickness of the STO layer, respectively, *e* is the electronic charge, and *E*_dep,th_ is the threshold *E*_dep_. Given that *ε* ~ 20*ε*_0_ of strained STO (Supplementary Fig. 7), ∆_bg_ = 3.2 eV and *t* = 3.9 nm, Eq. (3) yields *P*_th_ = 0.15 Cm^−2^, comparable to the value obtained in our simulation (Fig. 2c). At a certain AFM-tip loading force, therefore, the induced flexoelectric polarization could give rise to an abrupt increase in tunneling currents across ultrathin STO.

**Fig. 3:**
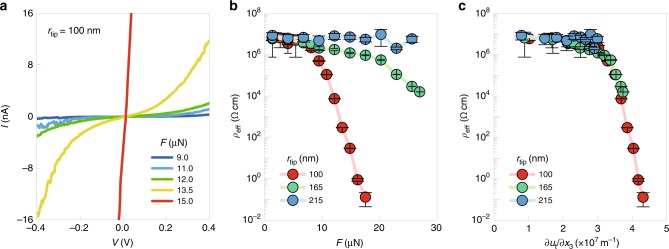
Colossal flexoresistance effect in ultrathin STO. **a** Current‒voltage (*I*–*V*) curves obtained by conductive AFM measurements in a 10-unit cell-thick STO film upon application of various tip loading forces *F*. Five representative curves are shown here. **b** Effective resistivity (*ρ*_eff_) as a function of *F*. **c** *ρ*_eff_ as a function of the AFM-tip-induced strain gradient ∂*u*_t_/∂*x*_3_. Error bars denote standard deviations of the fitted resistivity. Source data are provided as a Source data file.

Motivated by this, we use a conductive AFM tip to apply loading forces while simultaneously measuring the tunneling current (Fig. 2a). Conforming to the simulation condition, we use a diamond-coated tip with *r*_tip_ = 100 nm (Supplementary Fig. 8), which also can withstand much higher loading forces than other conductive tips (e.g., PtIr-coated tips). Figure 3a shows *I*–*V* curves measured at room temperature for a few representative loading forces (see Supplementary Fig. 9 for the entire set). At small applied forces (up to 7 μN), the measured current remains close to the noise level (a few pA). At intermediate applied forces (ranging from 7 to 13 μN), the *I*–*V* curves exhibit typical tunneling characteristics, and the current level increases gradually with the applied force. These results are ascribable to systematic modification of tunnel-barrier profiles under AFM-tip loading forces, consistent with our previous work^19^. Interestingly, when the applied forces exceed a threshold value (~15 μN), the *I*–*V* curves suddenly become linear in shape—characteristic of a highly conducting state. This highlights that the electrical state of ultrathin STO is switchable between highly insulating and conducting states, via purely mechanical means.

Importantly, this electrical-state switching in a large-bandgap dielectric naturally leads to an extremely large change in the electrical resistivity. During electrical-state switching, the effective resistivity of STO exhibits a colossal change with around eight orders-of-magnitude difference (Fig. 3b; see also Methods). Due to the detection limit of our equipment, we may underestimate the resistivity of the insulating state, i.e., 10^7^ Ω cm, compared with the bulk STO resistivity of over 10^9^ Ω cm; thus, the actual ratio of resistivity change could be larger than the estimated value. Given that we estimate the effective resistivity by taking into account the loading force dependence of the tip–STO contact area and STO thickness, we exclude any geometric anomaly as an origin for the observed effect. When we normalize the measured effect by applied pressures (i.e., loading forces divided by the tip–STO contact area), the relative increase in conductivity turns out to be as large as 10^−3^–10^−2^ Pa^−1^. Compared with other pressure-induced effects, such as piezoresistance (at most, 10^−7^ Pa^−1^)^26,27^, this effect not only shows several orders-of-magnitude enhancement, but also implies a distinctly new mechanism.

### Excluding other origins

**Fig. 4:**
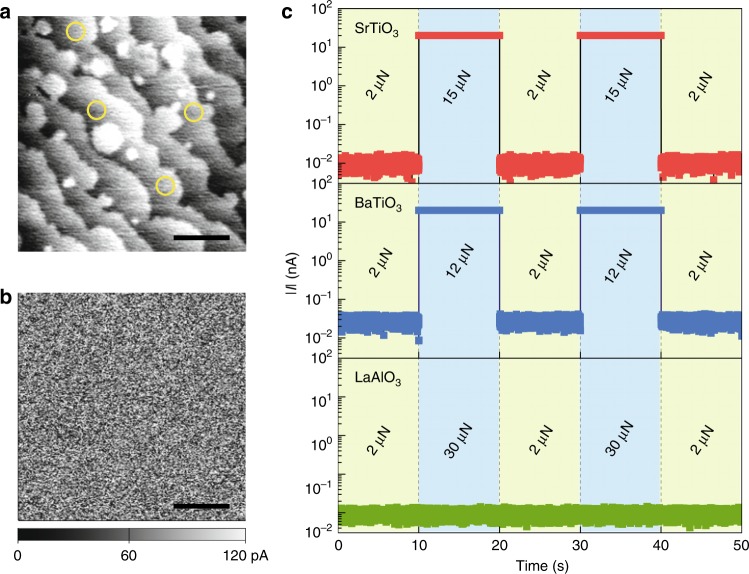
Reversible flexoresistance effect. **a** Topographical image obtained after the experiment. Regions where the experiments were conducted are marked by yellow circles. **b** Current mapping image on the same area, recorded with a 1-V bias voltage under a constant tip’s loading force of 2 μN, indicating that no current hotspot has been made after the experiment. **c** Current measured with a 0.1-V bias voltage under two representative loading forces in STO (red symbols), BaTiO_3_ (blue symbols) and LaAlO_3_ (green symbols). The lower threshold loading force (i.e., around 12 μN) for BaTiO_3_ may originate from inherently stronger flexocoupling strength in BaTiO_3_ (ref. ^29^), compared with that in STO. During the measurements, we set the current limit (compliance) to 20 nA. Scale bars in (**a**) and (**b**) represent 2 μm. Source data are provided as a Source data file.

Before addressing how flexoelectricity could explain our results, we rule out other possible origins of the phenomenon. We first exclude any involvement of an electrochemical process. The AFM-tip-induced mechanical force does not cause any permanent surface damage to the STO film (Fig. 4a, b), and the colossal control of resistance is reproducible, as proven by repeated exertion/withdrawal of the loading force (Fig. 4c). We also reproduce the same result even using a graphene top electrode (Supplementary Fig. 10). This again excludes any electrochemical interaction of STO with experimental environments, such as the AFM tip or ambient atmosphere, as graphene is impermeable to all atoms and molecules. In addition, based on quantitative and qualitative evidences (Supplementary Figs. 11–13), we exclude an electrostatic interaction between the AFM tip and STO as the primary origin of the colossal resistivity change observed.

Furthermore, the AFM-tip-induced strain itself cannot largely change the resistivity of STO that does not have *d* electrons. According to our theoretical analysis, the AFM-tip loading force generates compressive longitudinal strain *u*_33_ up to around 0.15 in STO (Supplementary Fig. 6). Because the antibonding and bonding states of Ti 3*d* and O 2*p* orbitals form the conduction and valence bands of STO, respectively, the compressive strain rather increases the bandgap of STO slightly (Supplementary Fig. 3)^23,24^; the effect of strain itself thus cannot explain the observed colossal decrease in STO resistivity, distinct from conventional piezoresistance effects^26,27^. For confirming this in our geometry, we repeat the experiments using AFM tips with different *r*_tip_ values of ~165 and ~215 nm (Supplementary Fig. 8). Although these tips can generate longitudinal strain *u*_33_, comparable to that by the tip with *r*_tip_ = 100 nm (Supplementary Fig. 6), the resulting resistivity changes are suppressed considerably (Fig. 3b and Supplementary Fig. 14). Therefore, these results suggest that the observed colossal decrease in resistivity could originate from AFM-tip-induced strain gradients, which modify the tunnel barrier via flexoelectricity.

### Strain-gradient-dependent resistivity change

Figure 3c indeed highlights the close correlation between the resistivity change and strain gradients. Strikingly, all of the data obtained with the three tips collapse to a nearly single curve when plotting the resistivity as a function of ∂*u*_t_/∂*x*_3_. This emphasizes the dominant contribution of ∂*u*_t_/∂*x*_3_ to the observed colossal reduction of resistivity. As predicted in Fig. 1, when the strain gradient-induced flexoelectric polarization reaches a threshold value, both the conduction band minimum and valence band maximum cross the Fermi level. This band crossing is capable of not only enhancing the tunneling conductance across STO (Fig. 1b) but also promoting interband tunneling between the STO valence and conduction bands, causing Zener breakdown^3,4,28^. Equations (2) and (3) estimate the threshold ∂*u*_t_/∂*x*_3_ required for the band crossing to be around 3 × 10^7^ m^−1^ for 10-unit-cell-thick STO, using ∆_bg_ = 3.2 eV, *t* = 3.9 nm, and *f*_eff_ = 25 V (ref. ^19^). This agrees quantitatively with our experimental results (Fig. 3c), in which the colossal decrease of resistivity begins at ∂*u*_t_/∂*x*_3_ ~ 3.5 × 10^7^ m^−1^. Taken together, our experimental and theoretical results consistently evidence that flexoelectric polarization-induced band crossing could explain the colossal reduction of resistivity, which we call the colossal flexoresistance.

### Colossal flexoresistance in various dielectrics

As such, it would be interesting to explore colossal flexoresistance in other dielectrics. As flexoelectricity is a universal phenomenon in all dielectrics, colossal flexoresistance could, in principle, be universal as well. Each dielectric, however, would require different threshold loading forces (i.e., threshold strain gradients) for colossal flexoresistance, depending on the inherent flexocoupling strength, bandgap, and so on. We repeat the experiments using BaTiO_3_, CaTiO_3_, and LaAlO_3_ of similar thicknesses (i.e., 10-unit-cell thick). For BaTiO_3_ and CaTiO_3_, we observe the same electrical-state switching (Fig. 4c and Supplementary Fig. 15), but at lower and higher threshold loading forces, respectively. The lower (or higher) threshold loading force for BaTiO_3_ (or CaTiO_3_) may originate from inherently stronger (or weaker) flexocoupling strength^29^ and/or smaller (or larger) bandgap, compared with those in STO. For LaAlO_3_, contrarily, we does not observe any noticeable resistance change up to the maximum AFM-tip loading force (Fig. 4c). LaAlO_3_ may have a much weaker flexocoupling strength due to its small Born effective charge^30^; additionally, its large bandgap (i.e., ∆_bg_ = 5.5 eV) also requires a large threshold polarization in Eq. (3). These conditions may make the threshold strain gradient in LaAlO_3_ too large to be achievable in our experimental geometry.

## Discussion

The colossal flexoresistance effect described here overcomes a long-standing dilemma: the electrical-state switching in dielectrics requires strong fields, but when applied by strong static fields, dielectrics inevitably suffer from irreversible damage. Utilizing universal flexoelectricity, we develop a general approach to apply non-destructive, strong electrostatic fields in various insulating systems, such as the Mott insulator^3^. Our approach will open up new avenues for unconventional quantum phenomena under strong static fields and device applications, such as the flexoelectronic transistor and mechanical sensor.

## Methods

### First-principles calculations

The atomic and electronic structures of the system were obtained using density functional theory (DFT) implemented in the Vienna ab initio simulation package (VASP)^31,32^. The projected augmented plane wave (PAW) method was used to approximate the electron–ion potential^33^. The exchange and correlation potentials were calculated using the local spin density approximation (LSDA). In the calculations, we employed a kinetic energy cutoff of 340 eV for PAW expansion, and a 6 × 6 × 1 grid of **k** points^34^ for Brillouin zone integration. The in-plane lattice constant was that of relaxed bulk STO (*a* = 3.86 Å); the *c*/*a* ratio and internal atomic coordinates were relaxed until the Hellman–Feynman force on each atom fell below |0.01| eV Å^−1^.

To understand the effect of electronic polarization on the interfacial electronic structure, we constructed a SrRuO_3_/STO bilayer with five unit cells of SrRuO_3_ and nine layers of STO, part of which is shown in Supplementary Fig. 1a. The sub-interfacial layers of the completely relaxed paraelectric phase of STO on SrRuO_3_ are insulating, and the Fermi level lies in the gap between the conduction band minima and valance band maxima. However, when STO is highly polarized, the induced electrostatic field largely bends bands; thus, both the conduction band minimum and valence band maximum of sub-interfacial STO layers could cross the Fermi level, as shown in the LDOS plot (Supplementary Fig. 1b). We plotted Supplementary Fig. 1b with frozen uniform displacement of the Ti atom by 0.18 and 0.54 Å. Note that polarized tetragonal STO has higher energy than paraelectric cubic STO, but can be stabilized under non-equilibrium strain conditions^35^. This band profile clearly supports the experimental finding that the band crossing of STO conduction and valence bands could lead to a colossal decrease in the electrical resistivity.

The dielectric constant was calculated using density functional perturbation theory^36–38^. Supplementary Fig. 7 represents the calculated total *zz* component of the total dielectric constant (i.e., *ε*_*zz*_), which includes both ionic and electronic contributions, as a function of strain *u*. The strain was measured with respect to the DFT equilibrium lattice of 3.86 Å.

In order to investigate the change in the bandgap of STO in the presence of the strain, we have used the hybrid functional (HSE06)^39^ implemented in the VASP package (Supplementary Fig. 3)^31,32^. We have used a 5-atom unit cell to simulate unstrained cubic and strained tetragonal structures in which an 8 × 8 × 8 **k**-point grid with energy cutoff of 600 eV are used. Convergence is reached if the consecutive energy difference is within 0.01 meV for electronic iterations and 0.1 meV for ionic relaxations. The calculated lattice constant of the cubic structure by full structure relaxation is 3.897 Å with a bandgap of 3.3 eV, in good agreement with experimental data^40,41^. For the strained tetragonal unit cell, a unit cell with compressive strain of 2 and 10% for *a* and *c* lattice constants (*a* = 3.819 Å, *c* = 3.507 Å) with respect to the relaxed cubic structure is considered based on the strain profile simulation. The calculated bandgap of the tetragonal structure is around 3.6 eV, slightly larger than that of the cubic structure.

### Wentzel–Kramers–Brillouin simulation

Using the one-dimensional WKB approximation, we can simply describe the tunneling current density for a low *T* and small *V*, as follows:

4 $$j(V) =	 \, \frac{{2e}}{h} \int_{ - \infty }^\infty {T(E)\, \times\, [f(E) - f(E - eV)]dE} \\ \cong 	\, {\frac{{2e}}{h}\int_{ - \infty }^\infty {\exp \left( - \frac{{4\pi }}{h} \int_0^d {\sqrt {2m(U(x) - E)} dx} \right)\, \times\, [f(E) - f(E - eV)]dE} }\\ \cong 	 \, {\frac{{2e}}{h}\exp \left( - \frac{{4\pi }}{h} \int_0^d {\sqrt {2m(U(x) - E_F)} dx} \right)\, \times\, eV},$$

where *T*(*E*), *f*(*E*), *U*(*x*), and *m* represent the transmission probability, Fermi–Dirac distribution, tunnel-barrier profile, and free electron mass, respectively. Using Eq. (4), we obtain the tunneling current density for a trapezoidal barrier profile (Supplementary Fig. 2) as follows^19^:

5 $$j( + V) =	\, \frac{{2e}}{h}\exp \left( - \frac{{4\pi }}{h} \int_0^{d_0} {\sqrt {2m\left\{ {\frac{{\varphi _2 - \varphi _1 + eV}}{{d_0}}(x - d_0) + \varphi _2} \right\}} dx} \right)\, \times\, eV \\ =	\, {\frac{{2e}}{h}\exp \left( - \frac{{8\pi \sqrt {2m} }}{{3h}} \cdot d_0 \cdot \frac{{(\varphi _2)^{1.5} - (\varphi _1 - eV)^{1.5}}}{{\varphi _2 - \varphi _1 + eV}}\right)\, \times\, eV},$$

6 $$j( - V) =	 - \frac{{2e}}{h}\exp \left( - \frac{{4\pi }}{h} \int_0^{d_0} {\sqrt {2m\left\{ {\frac{{\varphi _2 - \varphi _1 - eV}}{{d_0}}(x - d_0) + \varphi _2 - eV} \right\}} dx} \!\right) \times eV \\ =	 - {\frac{{2e}}{h}\exp \left( - \frac{{8\pi \sqrt {2m} }}{{3h}} \cdot d_0 \cdot \frac{{(\varphi _2 - eV)^{1.5} - (\varphi _1)^{1.5}}}{{\varphi _2 - \varphi _1 - eV}}\right) \times eV},$$

where *ϕ*_2_ and *ϕ*_1_ are the barrier heights of the right and left sides of the trapezoidal barrier, respectively, i.e., *ϕ*_1_ = *ϕ*_0_ + ∆*ϕ* and *ϕ*_1_ = *ϕ*_0_ − ∆*ϕ*. Using Eq. (4), we can also obtain the tunneling current density for a triangular barrier profile (Supplementary Fig. 2) as follows^19^:

7 $$j( + V) =	\, \frac{{2e}}{h}\exp \left( { - \frac{{4\pi }}{h} \int_0^{d{\prime}} {\sqrt {2m\left\{ {\frac{\varphi }{{d^{\prime}}}(x - d{\prime}) + \varphi } \right\}} dx} } \right) \times eV \\ =	\, {\frac{{2e}}{h}\exp \left( { - \frac{{8\pi \sqrt {2m} }}{{3h}} \cdot d \cdot \frac{{\varphi ^{1.5}}}{{\varphi + eV}}} \right) \times eV},$$

8 $$j( - V) =	 - \frac{{2e}}{h}\exp \left( { - \frac{{4\pi }}{h} \int_0^d {\sqrt {2m\left\{ {\frac{{\varphi - eV}}{d}(x - d) + \varphi - eV} \right\}} dx} } \right) \times eV \\ =	 - {\frac{{2e}}{h}\exp \left( { - \frac{{8\pi \sqrt {2m} }}{{3h}} \cdot d \cdot (\varphi - eV)^{0.5}} \right) \times eV},$$

where *ϕ* and *d* indicate the barrier height and width of the triangular barrier, and *d*′ = *d* · *ϕ*/(*ϕ* + *eV*). Importantly, depending on whether we consider the contribution of the STO valence band, *ϕ* and *d* have a different dependence on ∆*ϕ*. When neglecting the STO valence band, *ϕ* = *ϕ*_0_ + ∆*ϕ* and *d* = *d*_0_ · [(*ϕ*_0_ + ∆*ϕ*)/2∆*ϕ* (Supplementary Fig. 2a); this indicates that although the increased ∆*ϕ* reduces the barrier width *d*, it also increases the barrier height *ϕ*, such that the overall tunneling conductance cannot become largely enhanced (as shown in Fig. 1b; black dashed line). In striking contrast, when considering the STO valence band, *ϕ* is fixed to ∆_bg_ and *d* = *d*_0_ · *∆*_bg_/2∆*ϕ* (Supplementary Fig. 2b), which could lead to colossal enhancement of the tunneling conductance with increasing ∆*ϕ* (Fig. 1b).

### Sample fabrication

Ultrathin STO, BaTiO_3_, CaTiO_3_, and LaAlO_3_ films were grown by pulsed laser deposition, using a KrF excimer laser (*λ* = 248 nm). STO, BaTiO_3_, and LaAlO_3_ films were grown on bottom electrodes of epitaxial 20-nm-thick SrRuO_3_, prepared on TiO_2_-terminated and (100)-oriented STO substrates. CaTiO_3_ films were grown on LaAlO_3_ substrates buffered by LaNiO_3_ conducting electrode. The growth patterns and thickness were monitored by in situ reflection high-energy electron diffraction (RHEED; Supplementary Fig. 4). We deposited SrRuO_3_ and STO thin films at 700 °C under oxygen partial pressure of 100 and 7 mTorr, respectively. After deposition, films were annealed at 475 °C for 1 h in oxygen at ambient pressure and subsequently cooled to room temperature at 50 °C min^−1^. X-ray diffraction reciprocal space mapping was performed to ensure that the STO film was strain-free (Supplementary Fig. 5). Piezoresponse force microscopy found that as-grown BaTiO_3_ films had downward self-polarization (Supplementary Fig. 11).

### Simulation of strain profile

The strain distribution in ultrathin STO film pressed with an AFM tip is obtained by solving the elastic equilibrium equation in a 3D thin film/substrate system with appropriate boundary conditions. The detailed procedure is described in previous works^42^. Here, we discretized three-dimensional space into 100 × 100 × 500 grid points and applied periodic boundary conditions along the *x*_1_ and *x*_2_ axes. The grid spacing was ∆*x*_1_ = ∆*x*_2_ = 0.5 nm and ∆*x*_3_ = 0.1 nm. Along the *x*_3_ direction, 35 layers were used to mimic the film; the relaxation depth of the substrate featured 350 layers to ensure that the displacement was negligibly small. To estimate the surface stress distribution that developed with AFM-tip pressing, we adopted the closed-form solution derived by Wang et al. for indentation responses in a piezoelectric thin film in the ultrathin-film limit^43^. This contact mechanics model, comparing to the classical Hertz model for a non-piezoelectric, semi-infinite space^44^, considers not only the finite size of the film but also the coupled nature of the indentation problem of an electromechanically active sample. Therefore, it is more appropriate to describe the surface stress caused by nano-indentation in ultrathin STO films in the present work.

We considered an STO thin film of thickness *h*_*f*_, with the top surface in contact with an AFM tip and the bottom interface coherently constrained by the substrate. We assumed a conductive, rigid, spherical indenter with a tip radius *r*_tip_ = 100 nm and a mechanical force *F* ranging from 1 to 25 μN. At the top surface, the normal stress distribution (as a function of the distance from the contact center) is described as follows:

9 $$\sigma _{33}^{{\mathrm{tip}}}\left( r \right) = \left\{ {\begin{array}{*{20}{c}} { - c_{33}p\frac{{(a^2 - r^2)}}{{2h_{{\mathrm{def}}}}},}\hfill & {r \le a} \\ {0,}\hfill & {r \ge a} \end{array}} \right.,$$

where *a* is the contact radius $\Big(a = \sqrt {2R(h_{{\mathrm{ind}}} + \frac{{e_{33}}}{{c_{33}}}\phi _0)}\Big)$, *h*_ind_ is the indentation depth $\left(h_{{\mathrm{ind}}} = \sqrt {\frac{{Fh_{{\mathrm{def}}}}}{{c_{33}\pi R}}} - \frac{{e_{33}}}{{c_{33}}}\phi _0\right)$, *h*_def_ is the deformation depth *h*_def_ = *h*_*f*_, *c*_33_ is the elastic stiffness of the STO film (*c*_33_ = 336 GPa), *e*_33_ is the piezoelectric coefficient of the STO film (*e*_33_ = 0 Cm^−2^), and *ϕ*_0_ is the applied bias (*ϕ*_0_ = 0 V). At the film–substrate interface, the displacement is continuous for coherency and is assumed to relax to zero within a depth of *h*_*s*_ into the substrate (i.e., $\left. {\eta _i} \right|_{x_3 = - h_s} = 0$). The clamping effect of the STO substrate is considered to render the average strain zero at each layer of the film (i.e., $\overline {u_{11}} = \overline {u_{22}} = 0$ and $\overline {u_{12}} = 0$). Finally, the boundary value problem of elastic equilibrium, assuming no body force, is given by

10 $$\left\{ {\begin{array}{*{20}{c}} {\sigma _{ij,j} = 0}\hfill \\ {\left. {\sigma _{33}} \right|_{x_3 = h_f} = \sigma _{33}^{{\mathrm{tip}}},\left. {\sigma _{31}} \right|_{x_3 = h_f} = \left. {\sigma _{32}} \right|_{x_3 = h_f} = 0} \\ {\left. {\eta _i} \right|_{x_3 = - h_s} = 0}\hfill \end{array}} \right.,$$

where stress is related to strain via $\sigma _{ij} = c_{ijkl}e_{kl} = c_{ijkl}(u_{kl} - u_{kl}^0)$. The eigenstrain $u_{ij}^0$ is derived from strain-order parameter couplings of STO through $u_{ij}^0 = Q_{ijkl}P_kP_l + \lambda _{ijkl}q_kq_l$, where *Q*_*ijkl*_ and *λ*_*ijkl*_ are the electrostrictive and rotostrictive tensors, respectively. The electrostrictive and rotostrictive coupling coefficients of STO were adapted from ref. ^45^.

### Simulation of the polarization profile

The polarization distribution under the mechanical load by an AFM tip can be calculated by self-consistent phase-field modeling^46^. The temporal evolution of the polarization field **P**(**x**,*t*) is governed by the time-dependent Ginzburg–Landau equation, i.e., ∂**P**/∂*t* = − *L*(*δF*(**P**)/*δ***P**), where *L* is the kinetic coefficient and the total free energy *F* can be expressed as^46^

11 $$F =	 \, \int {\left( {f_{{\mathrm{bulk}}} + f_{{\mathrm{elastic}}} + f_{{\mathrm{electric}}} + f_{{\mathrm{gradient}}} + f_{{\mathrm{flexo}}}} \right)dV} \\ =	 {\int {\left[ {\alpha _{ij}P_iP_j + \alpha _{ijkl}P_iP_jP_kP_l + \beta _{ij}\theta _i\theta _j + \beta _{ijkl}\theta _i\theta _j\theta _k\theta _l + t_{ijkl}P_iP_j\theta _k\theta _l + \frac{1}{2}g_{ijkl}\frac{{\partial P_i}}{{\partial x_j}}\frac{{\partial P_k}}{{\partial x_l}}} \right.} } \\ 	{\left. { + \frac{1}{2}k_{ijkl}\frac{{\partial \theta _i}}{{\partial x_j}}\frac{{\partial \theta _k}}{{\partial x_l}} + \frac{1}{2}c_{ijkl}(u_{ij} - u_{ij}^0)(u_{kl} - u_{kl}^0) - \frac{1}{2}E_iP_i + \frac{1}{2}f_{ijkl}\left( {\frac{{\partial P_k}}{{\partial x_l}}\varepsilon _{ij} - \frac{{\partial \varepsilon _{ij}}}{{\partial x_l}}P_k} \right)} \right]dV}.$$

The bulk Landau free energy *f*_bulk_ consists of two sets of order parameters, i.e., the spontaneous polarization **P** and the antiferrodistortive order parameter **θ**, which represents the oxygen octahedral rotation angle of STO^45^. The flexoelectric contribution is considered as a Liftshitz invariant term as

12 $$f_{{\mathrm{flexo}}} = \frac{1}{2}f_{ijkl}\left( {\frac{{\partial P_k}}{{\partial x_l}}u_{ij} - \frac{{\partial u_{ij}}}{{\partial x_l}}P_k} \right).$$

The eigenstrain tensor ***u***^0^ in the elastic energy density is given by

13 $$u_{ij}^0 = Q_{ijkl}P_kP_l + \Lambda _{ijkl}\theta _k\theta _l - F_{ijkl}P_{k,l},$$

where the electrostrictive, rotostrictive, and converse flexoelectric couplings are considered via tensors **Q**, **Λ**, and **F**. The coefficients used in constructing the total free energy *F* of an STO single crystal are given in our previous works^45,47^. The transverse flexoelectric constant of STO estimated from experiments in the previous work was used (*f*_12_ = 25 V)^19^; the other two flexoelectric components were assumed to be zero (i.e., *f*_11_ = *f*_44_ = 0) for simplicity.

### Tunneling measurements

The *I*–*V* curves were obtained using an Asylum Research Cypher AFM (Oxford Instruments, Abingdon, UK) at room temperature under ambient conditions. Conducting diamond-coated metallic tips (DDESP-V2; BRUKER, Billerica, MA, USA) with nominal spring constants 80 Nm^−1^ and a dual-gain ORCA module (Oxford Instruments) were used to measure currents. In order to estimate the *r*_tip_ from the measured scanning electron microscopy (SEM) images (Supplementary Fig. 8a–c), we digitized the profile of the tip shape using a Java-based software (plot digitizer 2.6.8). The outline of the tip was tracked down with a scale of a pixel (~35 nm) in SEM images. Digitized data points were fitted with parabolic function ∆*z* = *c*_2_(∆*x*)^2^ + *c*_1_(∆*x*) + *c*_0_ (Supplementary Fig. 8d–f), then converted into *r*_tip_ as *r*_tip_ = 1/|2*c*_2_|.

An electrical bias was applied through the conducting SrRuO_3_ electrode; this was swiped from ‒0.5 to +0.5 V at a ramping rate of about 4 Vs^−1^. During the measurements, we set the current limit (compliance) to 20 nA. The noise floor of the AFM system was a few pA. We measured the resistance *R* from the linear slope of *I*–*V* curves in the low-bias regime. We extracted the resistance of STO, i.e., *R*_STO_, from the difference between the measured *R* and the resistance of the bottom SrRuO_3_ layer (i.e., ~70.4 kΩ; Supplementary Fig. 9i). We then estimated an effective resistivity (*ρ*_eff_) of STO by considering the effective tip–STO contact radius (*a*) and the effective STO thickness (*t*_STO_):

14 $$\rho _{{\mathrm{eff}}} = R_{{\mathrm{STO}}} \cdot \frac{{\pi a^2}}{{t_{{\mathrm{STO}}}}},$$

where we obtained the values of *a* and *t*_STO_ from our theoretical contact mechanics analysis.

### Graphene

For the graphene transfer onto the ultrathin BaTiO_3_ film, we followed the so-called dry-transfer technique. A graphene monolayer was mechanically exfoliated on a silicon wafer coated with poly(vinyl alcohol) (PVA), which is water-soluble, and poly(methyl methacrylate) (PMMA). After the selection of a proper graphene flake, the flake/PMMA layer was detached from the silicon substrate by immersion in hot deionized water. Then, the flake/PMMA layer floating on the water was transferred to a holder and was placed on the ultrathin BaTiO_3_ film using a homemade micromanipulator after alignment under an optical microscope. At last, the PMMA was removed with acetone.

## Supplementary information

Supplementary Information

Peer Review File

## Data Availability

All relevant data presented in this paper are available from the authors upon reasonable request. The source data underlying Figs. 1–4 and Supplementary Figs. 5–11,13–15 are provided as a Source data file.

## Source data

Source Data
