# Supplementary material for: Colossal flexoresistance in dielectrics
Source: Nat Commun. 2020 May 22;11:2586. doi: 10.1038/s41467-020-16207-7 (PMC7244591; doi:10.1038/s41467-020-16207-7)
Supplement: Supplementary file 1 — Supplementary Information [file 41467_2020_16207_MOESM1_ESM.pdf]

Supplementary Information

“Colossal flexoresistance in dielectrics”

Sung Min Park et al.

Supplementary Figures

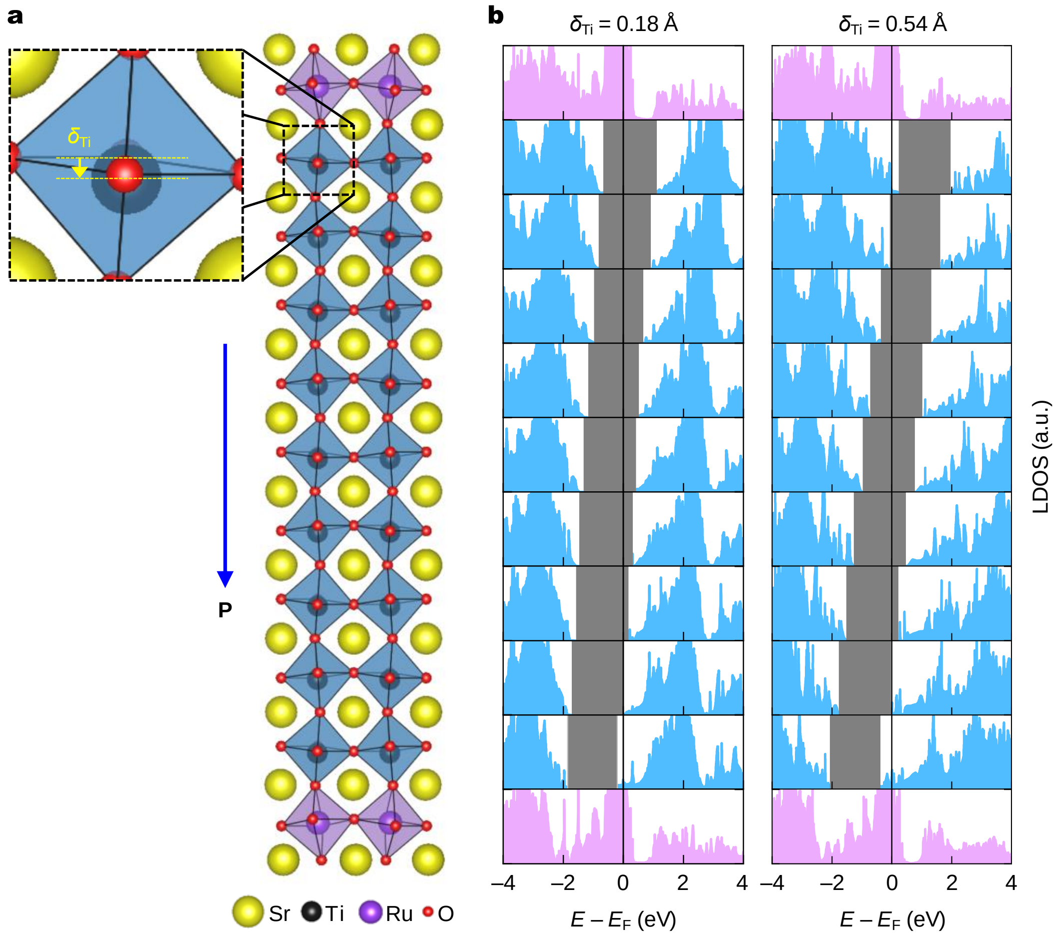

Supplementary Figure 1. Polarization-induced band crossing of STO conduction and valence bands. a, The simulation cell. We artificially polarized STO layers with uniform displacement of a Ti atom by δ_{Ti}. **b**, Calculated layer-resolved density of states (LDOS; filled blue) of polarized STO layers with δ_{Ti} = 0.18 Å (left) and 0.54 Å (right). Gray regions represent a gap between the conduction band minimum and valence band maximum of polarized STO layers, clearly showing a shift in the energy bands due to a polarization-induced electric field.

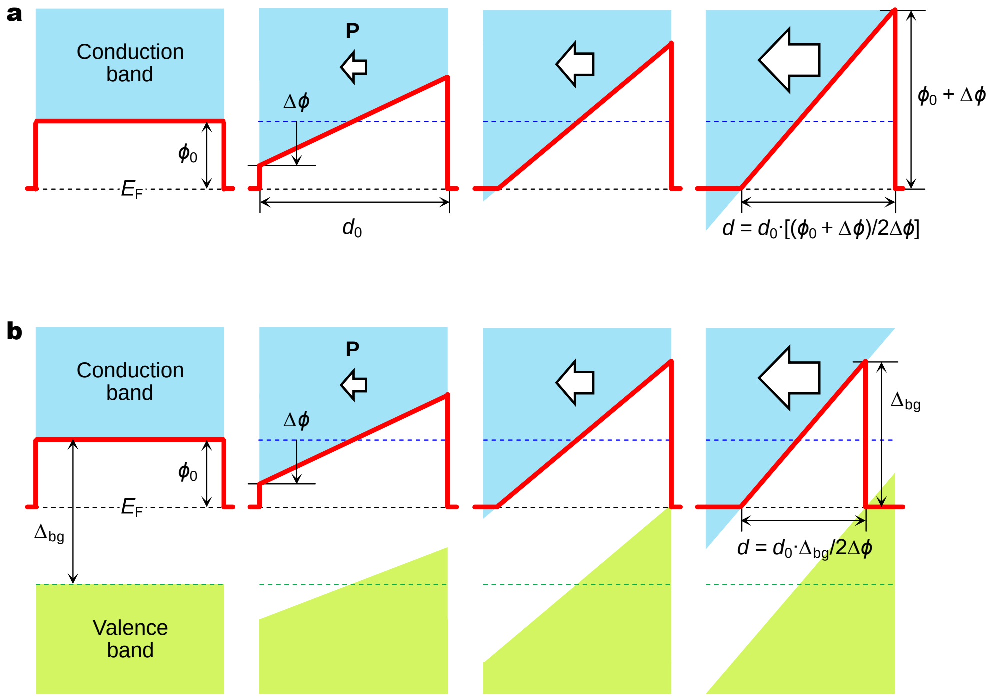

Supplementary Figure 2. Schematic diagram of the potential energy profiles across polarized STO. a,**b**, Red solid lines indicate the effective tunnel barrier with increasing flexoelectric polarization (**P**; white arrow) without (**a**) and with (**b**) considering the contribution of the STO valence band. Black dashed lines indicate the Fermi level. Blue and green dashed lines indicate the conduction band minimum and valence band maximum for **P** = 0, respectively.

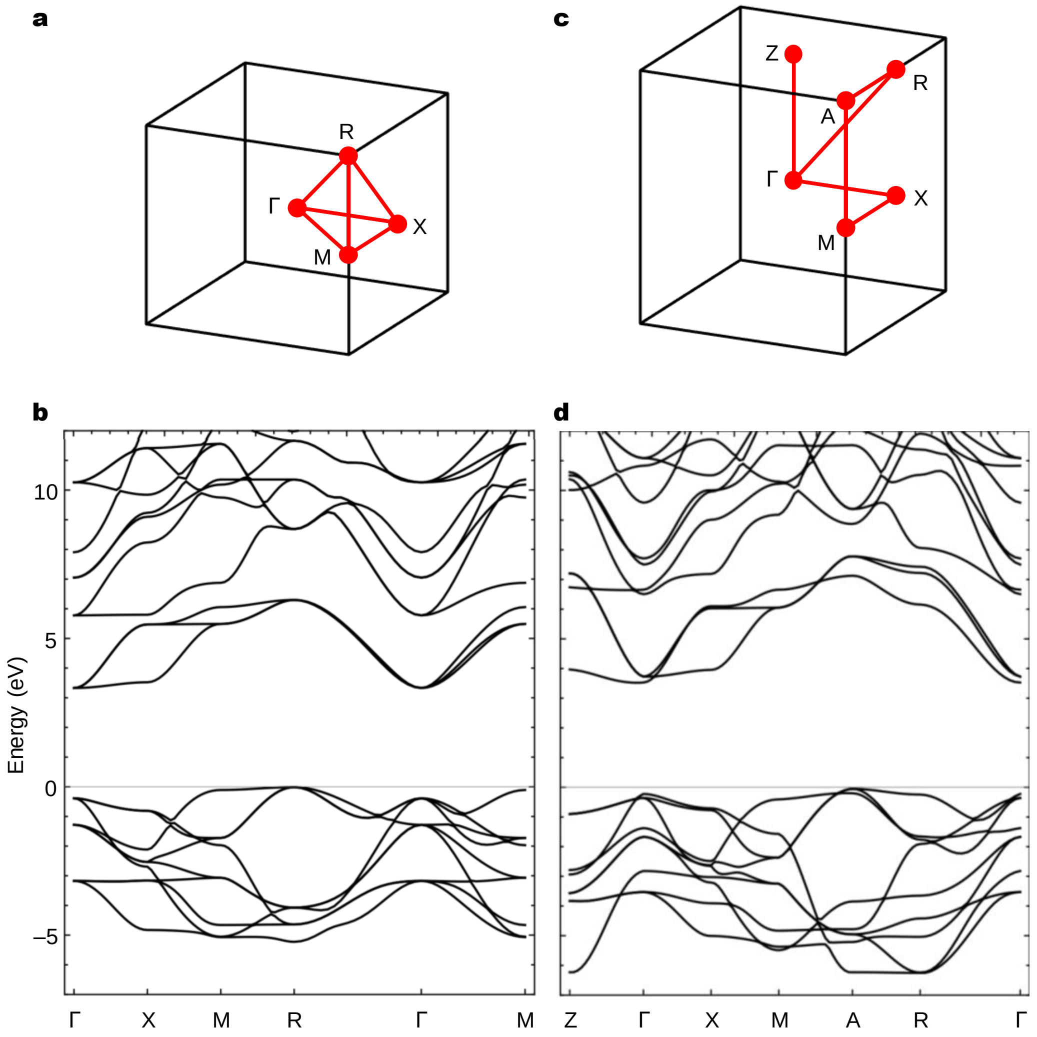

Supplementary Figure 3. Negligible strain effect in the STO bandgap. a,**b**, The Brillouin zone of a simple cubic STO ($a = 3.897$ Å) with the high-symmetry points (**a**) and corresponding band structure along the high-symmetry lines (**b**). **c**,**d**, The Brillouin zone of a strained tetragonal STO ($a = 3.819$ Å, $c = 3.507$ Å) with the high-symmetry points (**c**) and corresponding band structure along the high-symmetry lines (**d**). The zero of energy is set at the valence-band maximum.

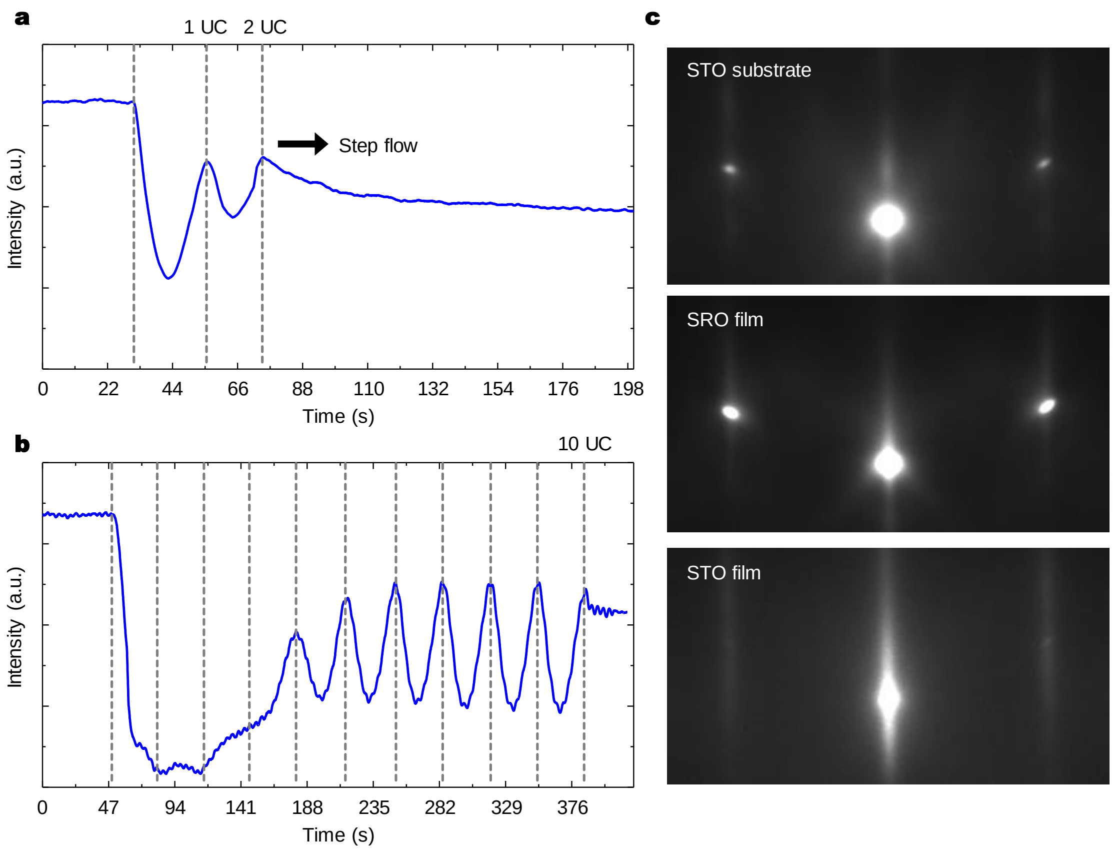

Supplementary Figure 4. Growth and characterization of ultrathin homoepitaxial STO. a,b Reflection high energy electron diffraction (RHEED) oscillations obtained during the growth procedure of (**a**) $SrRuO_3$ (SRO) and (**b**) STO films via pulsed laser deposition method, indicating the growth mode and the thickness of the films. **c**, *In-situ* reflection high energy electron diffraction of STO substrate, SRO film and STO film.

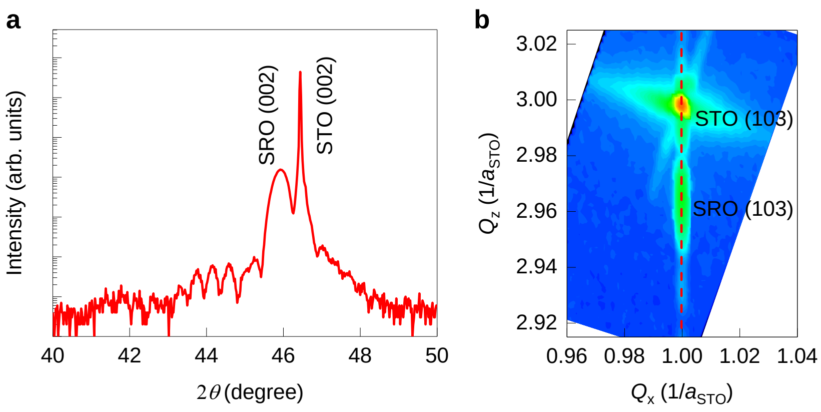

Supplementary Figure 5. Structural characterizations of $SrTiO_3$ thin films. a, X-ray diffraction (XRD) 2*θ*–*ω* scan of an $SrTiO_3$ (STO) thin film grown on a (001)-oriented STO substrate, with a conductive $SrRuO_3$ (SRO) buffer layer. The diffraction peak of SRO is indexed in pseudocubic perovskite notation. **b**, XRD reciprocal space mapping measured from the STO/SRO/STO (001) film around (103) diffraction. The red dashed line indicates that SRO thin film is fully strained on the STO (001) substrate. SRO (103) and STO (103) diffractions share the same in-plane lattice constant as 3.905 Å. Source data are provided as a Source Data file.

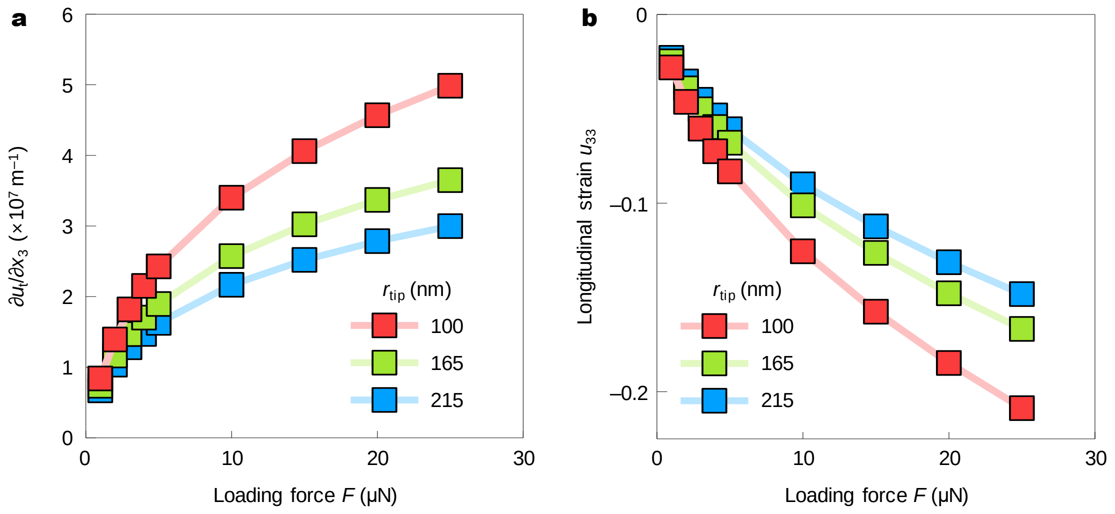

Supplementary Figure 6. Calculated strain gradients and strains imposed by the AFM tip. a, The averaged transverse strain gradients $\partial u_t/\partial x_3$ under the tip as a function of loading force. **b,** The averaged longitudinal strains u_{33} under the tip as a function of loading force. Source data are provided as a Source Data file.

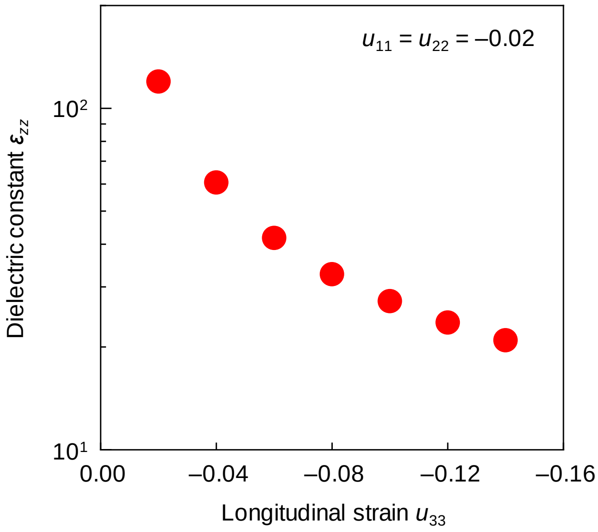

Supplementary Figure 7. Calculated dielectric constants. Density functional theory (DFT) calculations on the total *zz* component of the total dielectric constant (i.e., ε_{zz}), which includes both ionic and electronic contributions, as a function of longitudinal strain u_{33}. We fixed the transverse strains u_{11} and u_{22} to −0.02. The strain was measured with respect to the DFT equilibrium lattice of 3.86 Å. Source data are provided as a Source Data file.

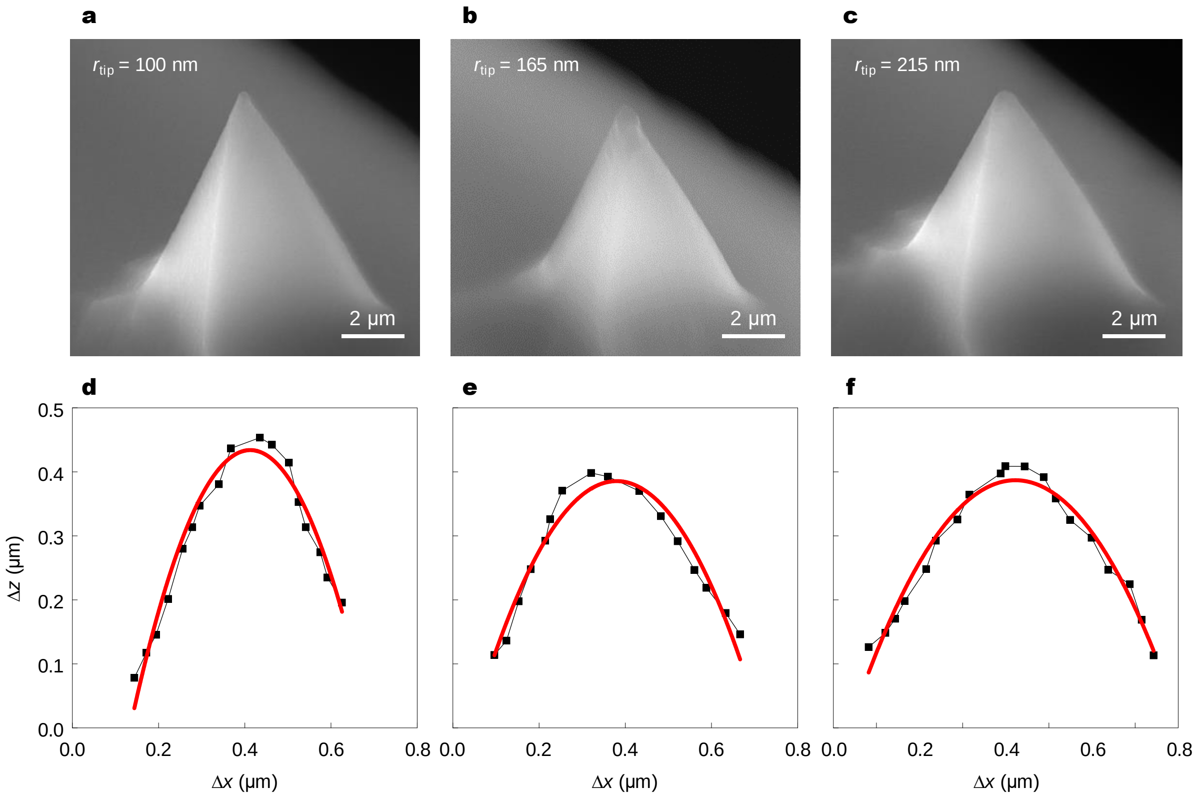

Supplementary Figure 8. Microscopic images of AFM tips. a–**c**, Scanning electron microscopy images of diamond-coated AFM tips with an r_{tip} estimated to be around 100 nm (**a**), 165 nm (**b**) and 215 nm (**c**). **d**–**f**, We digitized the profile of the tip shape (black squares), which we fitted with parabolic function $\Delta z = c_2(\Delta x)^2 + c_1(\Delta x) + c_0$ (red solid lines), where c_2, c_1, and c_0 are coefficients. From this, we estimated the radius of curvature r_{tip} as $r_{\mathrm{tip}} = 1/|2c_2|$. Source data are provided as a Source Data file.

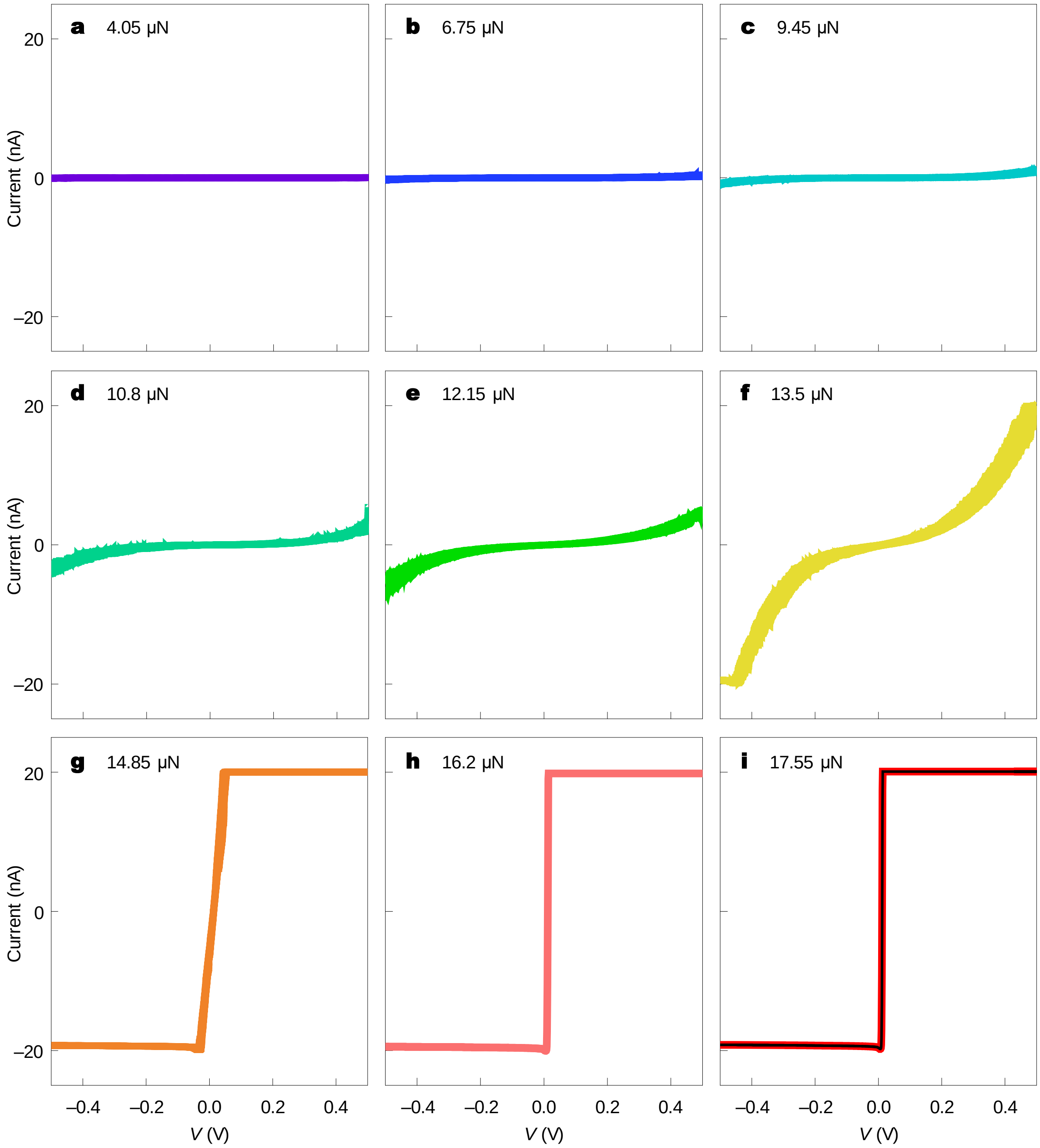

Supplementary Figure 9. Tunneling currents across a 10 unit cell-thick STO film. a–i, Tunneling currents with increasing forces. Before the critical force, *I*–*V* curves show tunneling like behavior and the magnitude increases gradually as the applied force increases. After the critical force, *I*–*V* curves show linear-like behavior. During the measurements, we set the current limit (compliance) to 20 nA. A black solid line in **i** indicates the *I*–*V* curve measured for a bare $SrRuO_3$ thin film, based on which we estimate the resistance of the bottom $SrRuO_3$ layer to be ~70.4 kΩ. Source data are provided as a Source Data file.

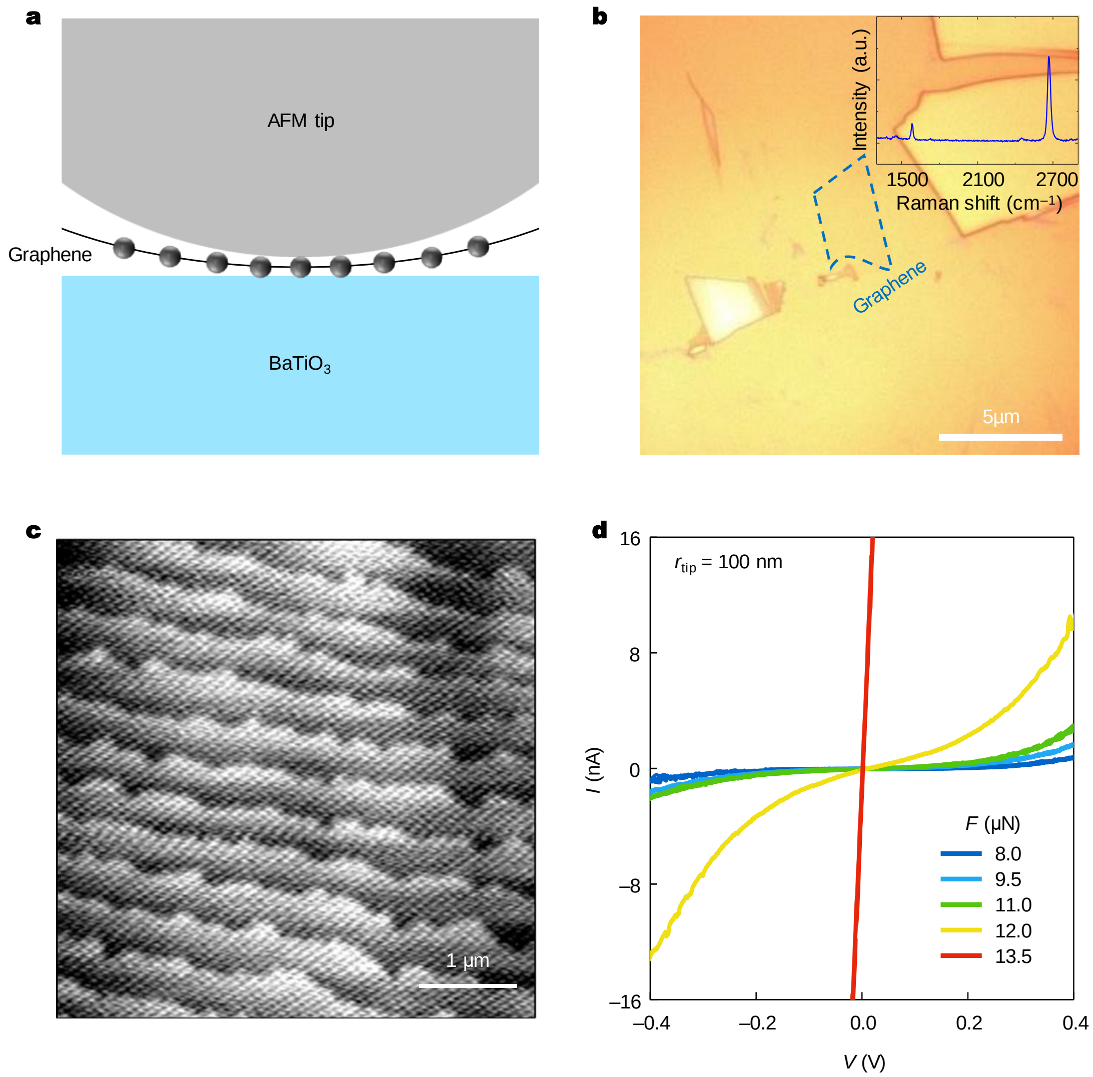

Supplementary Figure 10. Colossal flexoresistance in a 10 unit cell-thick $BaTiO_3$ film, buffered with a graphene. **a**, A schematic of experimental geometry with the graphene-buffered $BaTiO_3$ (Gr/BTO) film. **b**, An optical microscopy image of the Gr/BTO sample surface. Inset corresponds to Raman spectrum of an exfoliated graphene indicating mono-layer. **c**, An atomic force microscopy image of the Gr/BTO sample surface. **d**, Tunneling currents across the Gr/BTO sample with increasing tip loading forces. Source data are provided as a Source Data file.

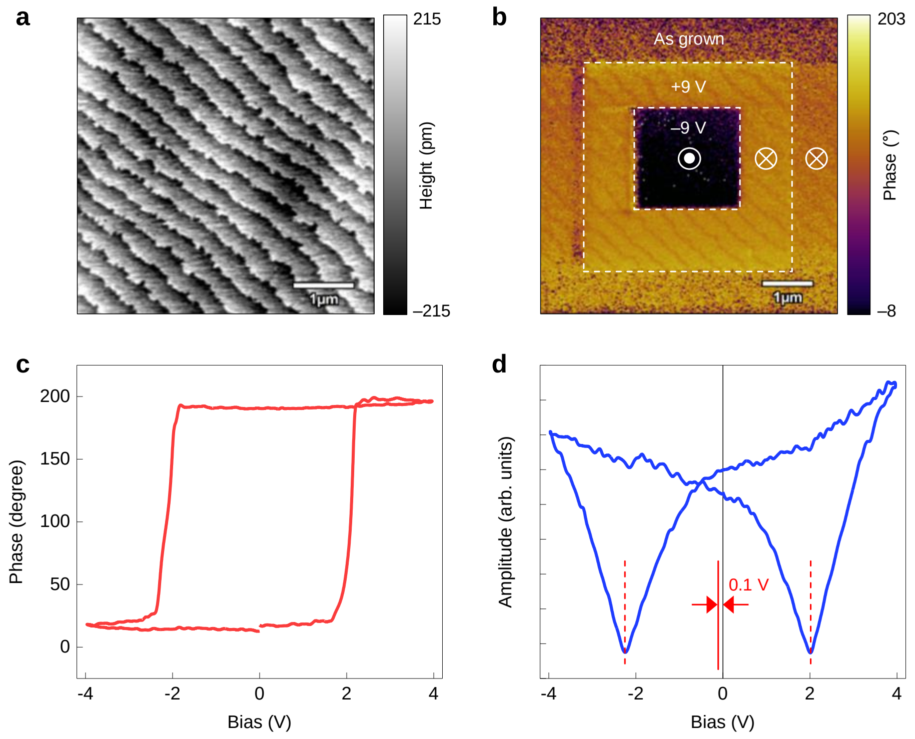

Supplementary Figure 11. Piezoresponse force microscopy (PFM) studies of $BaTiO_3/SrRuO_3/SrTiO_3$ heterostructure. a, Surface topography image of 10 unit cell-thick $BaTiO_3$. **b**, PFM phase images after electric bias applied. Black and yellow regions correspond to the region with upward and downward polarizations, respectively. **c**,**d**, PFM phase (**c**) and amplitude (**d**) hysteresis loops. We measure PFM hysteresis loops using a sufficiently low loading force. We observe that the PFM hysteresis loops are shifted by around 0.1 V. From this shift, we estimate the built-in electrostatic field in our experimental geometry to be at most 3 × 10^7 V m^{-1}. However, the colossal decrease in resistivity requires a much larger threshold electric field $E_{th} \sim \frac{\Delta_{bg}}{e \cdot t} \sim 8 \times 10^8$ V m^{-1} [Eq. (3) of the main text], where Δ_{bg} and t represent the bandgap and thickness of the dielectric layer, respectively. Therefore, the electrostatic interactions between the AFM tip and thin film cannot explain the observed colossal resistance change. Source data are provided as a Source Data file.

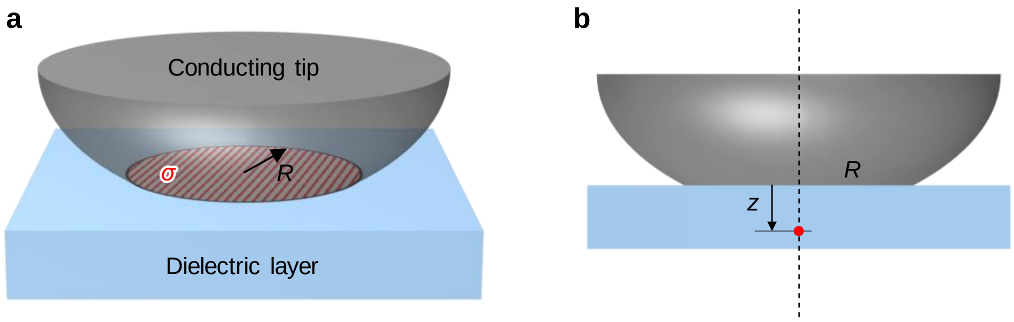

Supplementary Figure 12. Rough estimation of the out-of-plane electric field, generated by a surface charge of the AFM tip. a,**b**, The surface charge of the AFM tip, if any, can generate an electric field, thereby polarizing the dielectric layer. To check this possibility, we approximately estimate the electric field generated by a charged AFM tip. Here, we simplify the situation, and then consider the electric field generated by a uniformly charged circular disk with radius R, as in **a**. For this finite-sized charged disk, the out-of-plane electric field will decrease in magnitude as R decreases. For instance, a simple electrostatic calculation yields the out-of-plane electric field at a distance z from the center [i.e., dashed line in **b**] as $E = \frac{\sigma}{\varepsilon}[1 - \frac{z}{(z^2 + R^2)^{1/2}}]$, where σ is the surface charge density of the circular disk and ε is the dielectric permittivity of the dielectric layer. Since the contact radius R decreases with decreasing r_{tip}, the AFM tip with a smaller r_{tip} should induce a smaller out-of-plane electric field, thereby reducing the resistance change. (We assume that σ remains the same, not depending on r_{tip}.) However, this is opposite to our experimental observations. We observed colossal resistance change only in the case of using a sharp AFM tip (with a small r_{tip}). Therefore, the electrostatic interactions between the AFM tip and thin film cannot explain the observed r_{tip}-dependence of the resistance change.

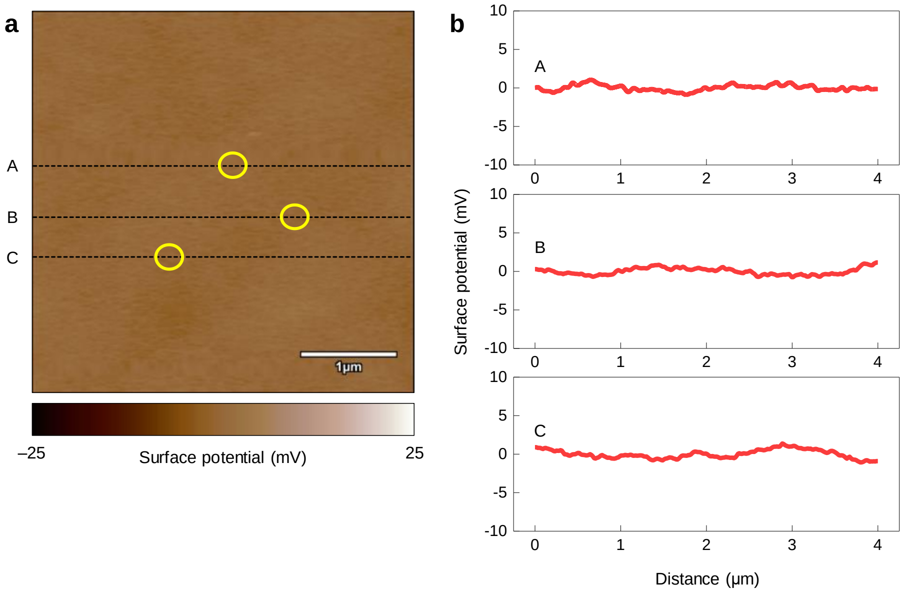

Supplementary Figure 13. Kelvin probe force microscopy (KPFM) studies. a, KPFM image showing surface potential profiles of $SrTiO_3$, after applying AFM tip loading force (~15 μN). Regions where the mechanical loadings were applied are marked by yellow circles. **b**, Line profiles of the surface potential, measured along the paths A, B, and C, denoted by dashed lines in **a**. The KPFM experiments showed almost no change in the surface potential (directly related to the surface charge state), indicating that the mechanical loading in our experiments did not induce or change the surface charge. Thus, the triboelectric effect cannot be the primary reason for the huge resistance change observed.

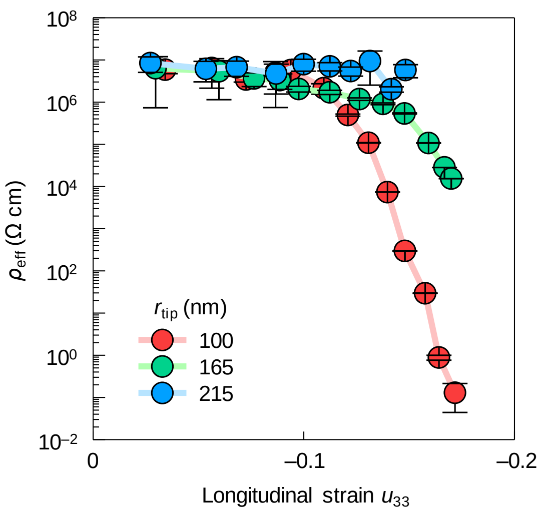

Supplementary Figure 14. Strain dependence of the resistivity. Although the tips with r_{tip} = 165 and 215 nm can generate strain, comparable to that by the tip with r_{tip} = 100 nm, the resulting resistivity changes are suppressed considerably. Error bars denote standard deviations of the fitted resistivity. Source data are provided as a Source Data file.

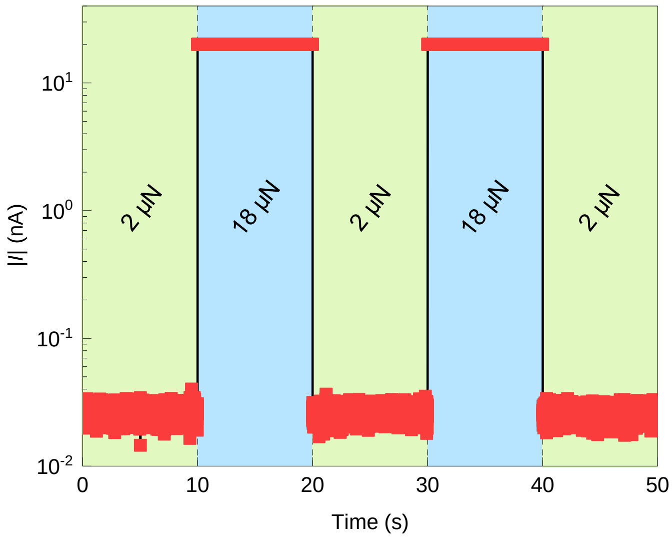

Supplementary Figure 15. Reversible flexoresistance behavior in 10 unit cell-thick $CaTiO_3$ film. Current measured with a 0.1-V bias voltage under two representative loading forces in $CaTiO_3$. The higher threshold loading force (i.e., around 18 μN) may originate from larger bandgap (i.e., ~3.8 eV) of $CaTiO_3$, compared to those (i.e., ~3.2 eV) of STO and $BaTiO_3$. During the measurements, we set the current limit (compliance) to 20 nA. Source data are provided as a Source Data file.

Supplementary Notes

Supplementary Note 1. Difference between earlier work and this work.

We utilized the depolarization field induced by flexoelectric polarization, in order to modify the tunnel barrier profile of an ultrathin dielectric in this work and previous work[1]. Also, for generating a flexoelectric polarization, we adopted the AFM tip-based experimental method in both the previous and current works.

However, the current work is distinctly different from the previous work[1], in terms of its purpose and achievement. Our previous work[1] focused mainly on how to estimate the flexocoupling coefficient at the nanoscale under high strain gradients. Then, we successfully demonstrated an effective way for characterizing nanoscale flexoelectricity under high strain gradients. Importantly, in our previous work, we mainly considered the regime of flexoelectric polarization, where a dielectric layer remains insulating. On the other hand, the current work focuses on how to achieve static, damage-free control of electrical states in dielectrics, which has remained a great challenge. For doing this, we considered the regime of large flexoelectric polarization, where the conduction and valence bands of $SrTiO_3$ could cross each other. As discussed in our manuscript, under these conditions, the “whole” $SrTiO_3$ layer behaves as a conductor, due to the highly enhanced tunnel conductance and/or Zener breakdown. In this work, therefore, we for the first time demonstrated static, damage-free control of electrical states in an otherwise highly insulating dielectric.

Supplementary References

1 Das, S. *et al.* Enhanced flexoelectricity at reduced dimensions revealed by mechanically tunable quantum tunnelling. *Nat. Commun.* **10**, 537 (2019).
